# Automatisierte Surveillance und Risikovorhersage mit dem Ziel einer risikostratifizierten Infektionskontrolle und -prävention (RISK Prediction for Risk-stratified Infection Control and Prevention)

**DOI:** 10.1007/s00103-024-03882-w

**Published:** 2024-05-16

**Authors:** Michael Marschollek, Mike Marquet, Nicolás Reinoso Schiller, Joëlle Naim, Seven Johannes Sam Aghdassi, Michael Behnke, Sandra Ehrenberg, Tatiana von Landesberger, Martin Misailovski, Fabian Prasser, André Scherag, Dirk Schlueter, Antje Wulff, RISK PRINCIPE Study Group, Anna Thalea Hoogestraat, Antje Wulff, Fabian Prasser, Luis Alberto Peña Diaz, Christine Geffers, Matthias Gietzelt, Claas Baier, Dirk Schlüter, Julia Hermes, Tim Eckmanns, Martin Boeker, Friedemann Gebhardt, Dirk Busch, Anne-Katrin Andreeff, Martin Sedlmayr, Katja de With, Jannik Schaaf, Holger Storf, Meta Bönniger, Jörg Janne Vehreschild, Simone Scheithauer, Martin Misailovski, Nicolás Reinoso Schiller, Martin Kaase, Dagmar Krefting, Martin Wiesenfeld, Martin Dugas, Alexander Dalpke, Mathias Pletz, Mike Marquet, André Scherag, Miriam Kesselmeier, Susanne Müller, Danny Ammon, Tatiana von Landesberger, Tom Baumgartl, Alexander Mellmann, Christian Philipps, Claudia Maria Hornberg, Oliver Kurzai, Stefanie Kampmeier, Rüdiger Pryss, Mathias Pletz, Simone Scheithauer

**Affiliations:** 1 Peter L. Reichertz Institut für Medizinische Informatik der TU Braunschweig und der Medizinischen Hochschule Hannover, Medizinische Hochschule Hannover, Hannover, Deutschland; 2 Institut für Infektionsmedizin und Krankenhaushygiene, Universitätsklinikum Jena, Jena, Deutschland; 3 Institut für Krankenhaushygiene und Infektiologie der Universitätsmedizin Göttingen, Robert-Koch-Straße 40, 37075 Göttingen, Deutschland; 4 Institut für Hygiene und Umweltmedizin der Charité Berlin, Berlin, Deutschland; 5 Visualisierung und Visual Analytics, Universität zu Köln, Köln, Deutschland; 6 Medizininformatik, Berlin Institute of Health at Charité – Universitätsmedizin Berlin, Berlin, Deutschland; 7 Institut für Medizinische Statistik, Informatik und Datenwissenschaften (IMSID), Universitätsklinikum Jena, Jena, Deutschland; 8 Institut für Medizinische Mikrobiologie und Krankenhaushygiene, Medizinische Hochschule Hannover, Hannover, Deutschland; 9 Big Data in der Medizin, Department für Versorgungsforschung, Fakultät VI Medizin und Gesundheitswissenschaften, Carl von Ossietzky Universität Oldenburg, Oldenburg, Deutschland

**Keywords:** Blutstrominfektion, Nosokomiale Infektion, Surveillance, Vorhersage, Risikostratifizierung, Bloodstream infection, Nosocomial infection, Surveillance, Prediction, Risk stratification

## Abstract

Nosokomiale Infektionen stellen weltweit, aber auch in Deutschland eine enorme Belastung für Patient*innen, Beschäftigte im Gesundheitswesen, Angehörige und die Gesellschaft dar. Zentrale Aufgaben der Infektionsprävention sind die Erfassung und Bewertung von Infektionen mit dem Ziel, Präventionspotenziale und Risikofaktoren zu identifizieren, geeignete Maßnahmen zu ergreifen und schließlich zu bewerten. Aus Sicht der Infektionsprävention wäre es von großem Wert, wenn (i) das Erfassen der Infektionsfälle automatisiert werden könnte und wenn (ii) es möglich wäre, im Voraus besonders gefährdete Patient*innen und Patient*innengruppen zu identifizieren, die von spezifischen und/oder zusätzlichen Interventionen profitieren würden.

Um diese risikoadaptierte bzw. individualisierte Infektionsprävention zu erreichen, entwickelt das Forschungsprojekt RISK PRINCIPE auf der Grundlage standardisierter, großer Datenbestände Algorithmen und computergestützte Anwendungen, welche Fachwissen im Bereich der Infektionsprävention nutzen.

Im Rahmen des Projekts werden 2 Ziele verfolgt: a) die Entwicklung sowie Validierung eines semiautomatischen Surveillance-Systems für im Krankenhaus erworbene Blutstrominfektionen, prototypisch für nosokomiale Infektionen, und b) die Verwendung von umfangreichen Patient*innendaten aus verschiedenen Quellen zur Erstellung eines individuellen oder gruppenspezifischen Infektionsrisikoprofils.

RISK PRINCIPE baut auf das Zusammenbringen der Expertisen von Medizininformatik und Infektionsmedizin mit dem Fokus auf Hygiene und nutzt u. a. Informationen und Erfahrungen aus 2 Konsortien (HiGHmed und SMITH) der deutschen Medizininformatik-Initiative (MII), die bereits über 5 Jahre erfolgreich an infektionsmedizinischen Anwendungsfällen gearbeitet haben.

## Abstract (en)

Healthcare-associated infections (HCAIs) represent an enormous burden for patients, healthcare workers, relatives and society worldwide, including Germany. The central tasks of infection prevention are recording and evaluating infections with the aim of identifying prevention potential and risk factors, taking appropriate measures and finally evaluating them. From an infection prevention perspective, it would be of great value if (i) the recording of infection cases was automated and (ii) if it were possible to identify particularly vulnerable patients and patient groups in advance, who would benefit from specific and/or additional interventions.

To achieve this risk-adapted, individualized infection prevention, the RISK PRINCIPE research project develops algorithms and computer-based applications based on standardised, large datasets and incorporates expertise in the field of infection prevention.

The project has two objectives: a) to develop and validate a semi-automated surveillance system for hospital-acquired bloodstream infections, prototypically for HCAI, and b) to use comprehensive patient data from different sources to create an individual or group-specific infection risk profile.

RISK PRINCIPE is based on bringing together the expertise of medical informatics and infection medicine with a focus on hygiene and draws on information and experience from two consortia (HiGHmed and SMITH) of the German Medical Informatics Initiative (MII), which have been working on use cases in infection medicine for more than five years.

## Einleitung

Aus medizinischer Sicht stellen nosokomiale Infektionen eine der größten medizinischen Belastungen dar, auch in Ländern mit hohem Einkommen [1]. Die Prävention und Surveillance von nosokomialen Infektionen ist eine der wichtigsten Aufgaben für die Patient*innensicherheit und ist im Infektionsschutzgesetz (IfSG; § 23 Abs. 1) fest verankert sowie auch in den Aufgaben der Kommission für Krankenhaushygiene und Infektionsprävention (KRINKO) und in den einschlägigen Regeln und Guidelines der APSS (Actionable Patient Safety Solutions) – eine Organisation, die sich für die Verbesserung der Patient*innensicherheit einsetzt [2].

Die Prävention nosokomialer Infektionen ist eine komplexe Herausforderung, die nicht durch eine einzelne Maßnahme gelöst werden kann. Die erfolgreiche Bewältigung dieser umfassenden Problematik erfordert die Zusammenarbeit von klinischen Expert*innen für Infektionsprävention/Hygiene, Data Stewards (setzen Datenrichtlinien und -standards um, verwalten Daten) und anderen Fachkräften aus den Bereichen der Infektionsmedizin, Statistik, Epidemiologie und medizinischen Informatik.

Aktuelle krankenhausinterne Surveillance-Maßnahmen sind ressourcenintensiv und deshalb realistisch nur in einem rollierenden System auf adäquater Qualitätsebene umsetzbar, bei dem einzelne Bereiche und Stationen hintereinander eingeschlossen werden. (Teil‑)Automatisierte Surveillance-Systeme (Programme oder Apps), die aktuell bereits kommerziell angeboten werden, können manuelle Fehler reduzieren. Jedoch wird ihre breite Anwendung durch Heterogenität und vor allem die mangelnde Interoperabilität erschwert, also die Fähigkeit unterschiedlicher Systeme oder Applikationen, möglichst nahtlos auf der Basis gemeinsamer Datenmodelle zusammenzuarbeiten. Die Surveillance von nosokomialen Infektionen ist entscheidend für Verbesserungen der Patient*innensicherheit und wird von der Weltgesundheitsorganisation (WHO) als Schlüsselkomponente zur Infektionsprävention betrachtet [3].

## Status quo

In Deutschland sind nosokomiale Infektionen für etwa 15.000 Todesfälle pro Jahr mitverantwortlich und die Zahl der Todesfälle durch nosokomiale Infektionen pro 100.000 Einwohner ist höher als in der Europäischen Union (EU; 20,1 gegenüber 15,3; [4]). Eine nationale Studie, die 2016 im Rahmen der europäischen Prävalenzerhebung durchgeführt wurde, ergab für Deutschland in einer repräsentativen Stichprobe eine Punktprävalenz von 3,6 % für nosokomiale Infektionen [5, 6]. In Universitätskliniken ist die Prävalenz höher (6,2 %), insbesondere auf Intensivstationen (17,1 %).

Neben den negativen Folgen, die eine nosokomiale Infektion für die einzelnen Patient*innen haben kann, sind nosokomiale Infektionen mit hohen Ausgaben im Gesundheitswesen assoziiert [7–12]. Die medianen Krankenhauskosten für Patient*innen mit Zentralvenenkatheter-assoziierten Blutstrominfektionen sind im Vergleich zu Patient*innen ohne Blutstrominfektion deutlich höher (60.445 € gegenüber 35.730 €; [9]).

Durch die massive Fachkräftebindung der Hygiene‑/Infektionsexpert*innen in der aktuellen Situation besteht oft nur ein geringes Potenzial, gezielte Risikofaktoranalysen durchzuführen und daraufhin den Einsatz bekannter infektionspräventiver Maßnahmen zu verstärken. Dabei kann es sich sowohl um eine Intensivierung von Standardmaßnahmen als auch um eine Allokation zusätzlicher Maßnahmen mit der Erfordernis einer besonders kritischen Güterabwägung (unerwünschte Arzneimittelwirkungen, Kosten etc.) handeln [13, 14]. Zu den hochwirksamen Infektionspräventions- und Infektionskontrollmaßnahmen zur Vermeidung nosokomialer Infektionen gehören die Steigerung der Handhygiene-Compliance des Personals [15] und die adäquate Besetzung mit spezialisierten Hygienefachkräften [16].

## Zielsetzung

**Abb. 1:**
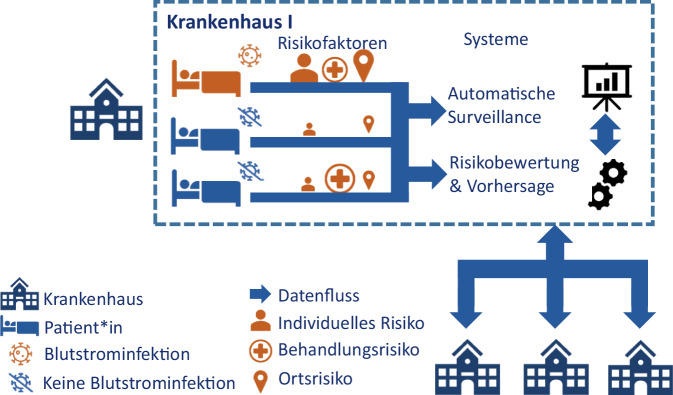
Schematische Darstellung der Informationsintegration im RISK-PRINCIPE-Projekt (eigene Abbildung)

Das vom Bundesministerium für Bildung und Forschung (BMBF) im Rahmen der Medizininformatik-Initiative (MII) geförderte Projekt RISK PRINCIPE (Risk-stratified Infection Control and Prevention) ist im Juli 2023 mit einer Kooperation aus 13 deutschen Universitäten, dem Robert Koch-Institut (RKI), dem Nationalen Referenzzentrum für Surveillance von nosokomialen Infektionen, dem Aktionsbündnis Patientensicherheit und den BG Kliniken – Klinikverbund der gesetzlichen Unfallversicherungen GmbH gestartet (Tab. 1). Ziele während der 4‑jährigen Laufzeit des Projekts sind die semiautomatisierte, computergestützte Surveillance sowie die datengetriebene Risikoanalyse und -vorhersage mit dem Ziel einer individualisierten, risikostratifizierten Infektionsprävention und -kontrolle (Abb. 1). Dadurch sollen die Qualität der Krankenversorgung sowie die Patient*innensicherheit verbessert werden.

**Tab. 1:** Übersicht der RISK-PRINCIPE-Projektpartner

Stadt	Institution(en)
Berlin	BG Kliniken – Klinikverbund der gesetzlichen Unfallversicherungen GmbH (Hauptsitz Berlin mit den Standorten Bad Reichenhall, Berlin, Bochum, Bremen, Duisburg, Frankfurt am Main, Halle, Hamburg, Ludwigshafen, Murnau, St. Peter-Ording, Tübingen), Charité – Universitätsmedizin Berlin, Robert Koch-Institut und das Nationale Referenzzentrum für Surveillance von nosokomialen Infektionen
Bielefeld	Universität Bielefeld
Bonn	Aktionsbündnis Patientensicherheit
Dresden	Universitätsklinikum Dresden
Frankfurt	Universitätsklinikum Frankfurt
Göttingen	Universitätsmedizin Göttingen
Hannover	Medizinische Hochschule Hannover
Heidelberg	Universitätsklinikum Heidelberg
Jena	Universitätsklinikum Jena
Köln	Universität zu Köln
München	Technische Universität München
Münster	Universitätsklinikum Münster
Oldenburg	Carl von Ossietzky Universität Oldenburg
Würzburg	Universitätsklinikum Würzburg

Im Projekt RISK PRINCIPE bearbeitet das Team 2 Hauptaufgaben: a) die Verwendung von Patient*innendaten zur Erstellung eines Patient*innen- bzw. Patient*innengruppen-spezifischen Risikoprofils, das in eine computergestützte Anwendung übertragbar ist, und b) die Entwicklung sowie Validierung einer automatisierten Datenerfassung für Surveillance-Zwecke und eine routinedatenbasierte Risikovorhersage am Beispiel von im Krankenhaus erworbenen Blutstrominfektionen (*Hospital-onset Bloodstream Infection*, HOB) mit anschließender Visualisierung.

Das Projekt basiert auf einer strategischen Allianz zwischen Medizininformatik, Surveillance und Infektionsprävention/Hygiene, die die Grundlage für eine dauerhafte und nachhaltige Zusammenarbeit bildet. Die Strukturierung und Durchführung des Projekts erfolgt in aufeinander abgestimmten und eng vernetzten Arbeitspaketen (AP), die von einer Governance-Struktureinheit koordiniert werden. Eine detaillierte Aufschlüsselung dieser Prozesse findet sich im folgenden Abschnitt.

## Arbeitspakete

**Abb. 2:**
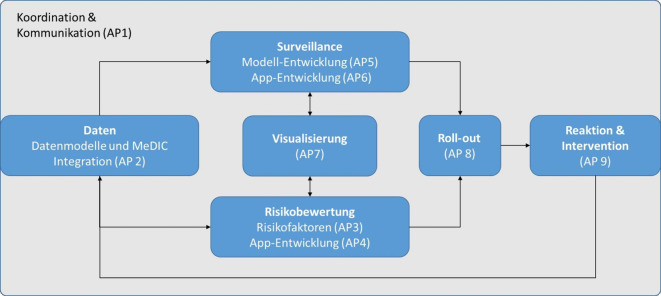
Darstellung der Struktur und Interaktion der Arbeitspakete (AP) im Projekt RISK PRINCIPE (eigene Abbildung). *MeDIC* medizinisches Datenintegrationszentrum

RISK PRINCIPE besteht aus 9 AP (Abb. 2), die in 4 Stufen eingeteilt werden:

1. Koordination, Governance, Vernetzung und Bewertung (AP1),
2. Datenstrukturierung, Integration und Identifikation von HOB-Risikofaktoren (AP2–AP3),
3. App- und Algorithmusentwicklung (AP4–AP6),
4. Visualisierung und Roll-out einer App (AP7–AP9).

*AP1* (Koordination, Governance, Vernetzung und Bewertung) konzentriert sich dabei hauptsächlich auf die Schaffung geeigneter Rahmenbedingungen, darunter die Einbindung der Ethikkommission, Entwicklung eines Datenschutzkonzepts sowie die Implementierung von Governance-Strukturen. Die Governance und Koordination erfolgen integriert in diesem AP, welches ein Kernteam (eine PI und drei Co-PIs mit ihrem Team) leitet.

Auch die Überwachung des Projektfortschritts anhand von Meilensteinen, die Evaluierung der Vorhersagekraft des Prototyps und des endgültigen Risikovorhersagemodells sowie die Prüfung der Benutzerfreundlichkeit sind essenziell für die Bewertung des Projekterfolgs.

Im Rahmen des Projekts sollen Routinedaten in die Datenplattformen der beteiligten medizinischen Datenintegrationszentren (MeDICs) überführt werden. Diese stellen die technischen und organisatorischen Voraussetzungen für die standortübergreifende Datennutzung zwischen Krankenversorgung und medizinischer Forschung dar.

In *AP2* liegt der Fokus auf der interoperablen Nutzbarmachung, also der Standardisierung der infektionsbezogenen Routinedaten, die bislang ausschließlich in den klinischen Primärdokumentationssystemen in oft sehr unterschiedlicher Form vorliegen. Dies sind die Daten, die im Krankenhaus durch das medizinische Personal für die Zwecke der Versorgung im Krankenhausinformationssystem erfasst und gepflegt werden. Für die Umsetzung müssen unter Berücksichtigung der international anerkannten FAIR-Prinzipen (engl. *Findable, Accessible, Interoperable, and Re-usable*) gemeinsam Zielstrukturen der zu nutzenden Daten vereinbart sowie Datenschutz und IT-Sicherheitsaspekte berücksichtigt werden.

Die Informations- und Datenmodelle des Kerndatensatzes der MII (www.medizininformatik-initiative.de) sind hierbei entscheidende Vorbilder. In diesem ist auf nationaler Ebene festgelegt, welche Daten von den MeDICs je Anwendungsfall in einer standardisierten Form mindestens vorzuhalten sind, sodass eine standortübergreifende Nutzung der Daten möglich ist. Bislang nicht abgedeckte Datenelemente (und neue primäre Datenquellen aus den Krankenhäusern) werden unter Verwendung standardisierter medizinisch-fachlich geprüfter Informationsmodelle (Struktur zur Datenorganisation und -repräsentation) spezifiziert und einheitlich gespeichert. Für die Spezifikation werden auch andere existierende Informationsmodelle wie das des Deutschen Zentrums für Infektionsforschung (DZIF), Ergebnisse einschlägiger Studien [17] und systematischer Literaturrecherchen (als Teile von AP3) sowie Meinungen von Fachexpert*innen im Hinblick auf die spezifischen Ziele von RISK PRINCIPE, aber auch für zukünftige Nutzungsszenarien herangezogen.

Standardisierte Informationsmodelle sind über die Institutionsgrenzen hinweg definierte, einheitliche Beschreibungen von Daten und zugehörigen Metadaten. Die Anpassung von heterogenen Daten aus unterschiedlichen Quellen in ein solches einheitliches Modell ist eine Voraussetzung dafür, um Daten in die MeDICs zu überführen und zwischen den Standorten austauschbar zu machen.

Darüber hinaus zielen die Arbeiten in AP2 auch darauf ab, aktuelle Lücken in den Primärdatenquellen zu identifizieren (z. B. in der Datenqualität). Ein besonderer Schwerpunkt liegt dabei auf Mikrobiologiebefunden und auf der Integration von Sequenzierungsdaten des Infektionserregers, um die Extraktion von virulenz- und resistenzbezogenen Informationen für eine spezifische Risikobewertung zu ermöglichen. In AP2 ist daher, wie in allen APs, eine schon im vorherigen *Use Case Infection Control* des Konsortiums HiGHmed initiierte und etablierte Tandembearbeitung (Tandemansatz) entscheidend, d. h. eine enge interdisziplinäre Zusammenarbeit zwischen Personen mit medizinisch-fachlicher Expertise (Datenentstehung, Datennutzung, Semantik) sowie informatisch-technischen Kenntnissen (Datenmodellierung, Interoperabilitätsspezifikation, Maschinenlernen).

Am Beispiel von HOB sollen die datengetriebene Risikoanalyse und -vorhersage mit dem Ziel einer individualisierten, risikostratifizierten Infektionskontrolle und -prävention etabliert werden. Dazu werden in *AP3* (Methoden zur individuellen Risikoanalyse und Vorhersagemodellierung) zuerst Risikofaktoren für HOB identifiziert sowie Vorhersagemodelle für HOB entwickelt.

Risikofaktoren für HOB lassen sich unterteilen in patient*innenspezifische, anwendungsspezifische und ortsspezifische Faktoren. Des Weiteren muss auch von erregerspezifischen Faktoren ausgegangen werden. Die genaue Identifizierung, Bewertung und Gewichtung dieser Risikofaktoren erfolgen anhand einer sorgfältigen Analyse externer Forschungsergebnisse, einer umfassenden Literaturrecherche sowie der Analyse eines umfangreichen und qualitativ hochwertigen eigenen Datensatzes aus der ALERTS-Studie [17] mit 62.154 eingeschriebenen Patient*innen und mehr als 3200 nosokomialen Infektionen. Diese Daten in Kombination mit klinischen Daten, die aktiv dokumentiert oder aus dem Krankenhausinformationssystem abgeleitet werden, sollen es ermöglichen, Risikofaktoren und Vorhersagemodelle für HOB retrospektiv zu identifizieren und das Verständnis für Infektionsrisiken in Krankenhäusern zu vertiefen.

Die entwickelten Modelle werden anschließend prospektiv an mehreren MeDICs im Rahmen des Projekts validiert. Das Risikomodell soll in der Lage sein, Risikoprofile für Patient*innenrisikogruppen zu erstellen. Im Laufe des Vorhabens sollen verschiedene *Machine-Learning*-Algorithmen zur Anwendung kommen, um bislang unbekannte Risiken und mögliche Muster in einem explorativen Ansatz zu identifizieren, die zur Verbesserung der Prävention von HOB beitragen können.

*AP4 *(digitale Unterstützung bei der Risikoanalyse und -vorhersage von Blutstrominfektionen) konzentriert sich auf die Entwicklung einer digitalen Demonstrator-Applikation (Testversion der Applikation) zur Unterstützung der Risikoanalyse und -vorhersage von HOB im klinischen Umfeld. Dafür arbeitet ein hochgradig interdisziplinäres Team aus Expert*innen der medizinischen Informatik, der Mikrobiologie sowie der Infektionsprävention/Hygiene in einem iterativen Prozess daran, spezifische Funktionen, Ansichten und Analysen in einer Anwendung umzusetzen. Der entworfene Demonstrator (App) soll medizinischem Personal wichtige Informationen für die Risikoeinschätzung zur Verfügung stellen, indem sowohl medizinische Routinedaten (bspw. Entzündungsparameter im Blut, mikrobiologische Befunde) aufbereitet und dargestellt als auch die in AP3 entwickelten algorithmischen Risikovorhersagemodelle integriert werden.

Die Ausgestaltung gründet sich hierbei auf eine umfangreiche Anforderungsanalyse, um die technischen und klinischen Notwendigkeiten passgenau abbilden zu können. Für die Gestaltung der grafischen Benutzeroberfläche werden die Ergebnisse des AP7 verwendet, um interaktive Visualisierungen zu ermöglichen. Das architektonische Design wird so ausgearbeitet, dass es den Bedürfnissen aller Partner (Aktionsbündnis Patientensicherheit, Nationales Referenzzentrum für Surveillance von nosokomialen Infektionen, BG Kliniken) entspricht. Die Applikation wird auf abgestimmten, offenen (frei verfügbaren) Datenmodellen aufbauen und standardisierte Schnittstellen für den Zugriff auf die in den MeDICs konsolidierten Routinedaten verwenden (siehe AP2). Über einen Roll-out-Prozess (AP8) soll die Anwendung allen teilnehmenden Universitätskliniken und dem RKI zur Verfügung gestellt werden. Die Demonstrator-Anwendung wird langfristig dazu beitragen, eine effektive und effiziente Infektionsprävention weiterzuentwickeln sowie das Krankenhauspersonal zu unterstützen.

Aktuell stellt die laut IfSG verpflichtende Surveillance nosokomialer Infektionen in deutschen Krankenhäusern, i. d. R. durch manuelle Erfassung von Fällen, einen zeitaufwendigen Arbeitsprozess dar. Für gewöhnlich werden die Ergebnisse mikrobiologischer Proben im Laborinformationssystem des jeweiligen Krankenhauses digital gespeichert und können potenziell durch spezifische Algorithmen abgefragt werden. Es besteht somit ein bislang ungenutztes Potenzial, den Arbeitsaufwand der Erfassung zu reduzieren und in der Surveillance vernachlässigte Versorgungsbereiche stärker zu berücksichtigen.

Durch die hohe Korrelation mit systemischen Infektionen stellen die HOB einen sinnvollen Anwendungsfall für eine semiautomatisierte Erfassung nosokomialer Infektionen dar. Die technische Machbarkeit konnte bereits in mehreren Ländern gezeigt werden [18]. In *AP5* (Methoden der semiautomatischen Surveillance) wird unter starker Berücksichtigung des in AP2 definierten Kerndatensatz-Subsets ein Algorithmus entwickelt, der eine semiautomatisierte Detektion von HOB-Fällen und die Bildung nach unterschiedlichen Kennzahlen stratifizierter HOB-Raten erlaubt. Die Spezifikationen des Algorithmus werden dabei mit dem internationalen PRAISE-Netzwerk (*Providing a Roadmap for Automated Infection Surveillance in Europe*; [19]) abgestimmt, womit Perspektiven für ein zukünftiges europäisches Benchmarking der erhobenen Daten geschaffen werden.

Neben den eigentlichen Risikovorhersagemodellen ist eine übersichtliche und einfach verständliche Ergebnisübersicht zur Ergebnisinterpretation essenziell. In *AP6 *(digitales Tool für die semiautomatische Surveillance von HOB in Krankenhäusern) soll dazu ein interaktives Dashboard entwickelt werden, mit dem Anwender*innen dynamische Indikatoren und Krankenhausbereiche auswählen können, um die dazugehörigen Daten auszuwerten und zu monitoren. Das Dashboard wird, ausgehend von einer umfangreichen Anforderungsanalyse, gemeinsam durch die verschiedenen Projektpartner in einem iterativen Prozess entwickelt [20]. Dieser Ansatz soll einen Fokus auf die Verfeinerung des webbasierten Dashboards und die darin enthaltenen Visualisierungen ermöglichen.

Der im Rahmen von RISK PRINCIPE entwickelte und in die Anwendung integrierte Algorithmus soll zusätzlich zur Darstellung der hausinternen Daten auch die semiautomatische Erkennung von potenziellen HOB-Risikofaktoren ermöglichen. Die Informationen, welche die Anwendung darstellt, werden aus dem MII-Kerndatensatz abgeleitet [21]. Eine Bereitstellung erfolgt durch die MeDICs der Standorte. Die Basisversion der Anwendung soll im Anschluss um die Möglichkeit ergänzt werden, standortübergreifende Referenzdaten im Dashboard darzustellen und für vergleichende Analysen zu nutzen.

Neben AP6 konzentriert sich *AP7 *(Entwicklung interaktiver Visualisierungen zur Risikoanalyse und Surveillance in der Infektionskontrolle der HOB) auf die Entwicklung einer interaktiven Visualisierung, die das Verständnis von HOB-Risikofaktoren, deren Identifikation und Erforschung ermöglicht. Eine benutzerfreundliche Visualisierung soll eine einfache und schnelle Interpretation der Ergebnisse ermöglichen, insbesondere bzgl. der Identifikation und des Beitrags von einzelnen Risikofaktoren zum Gesamtrisiko. Die Visualisierungsoberfläche stellt verfügbare und abgeleitete Risikofaktoren sowie Modellergebnisse interaktiv dar und enthält auch Vorhersagen.

Die Herausforderung besteht darin, geeignete visuelle Designs zu entwickeln, die den Anforderungen der Nutzer*innen und den komplexen Daten und Modellen gerecht werden. Die enge Zusammenarbeit mit klinischen Partner*innen ist erforderlich, um eine effektive und bedienungsfreundliche *User Experience* zu gewährleisten. Dies umfasst die Anforderungserhebung und Datencharakterisierung in Zusammenarbeit mit AP2 und Expert*innen der Infektionskontrolle und Hygiene, das Design und die Umsetzung zur Risikofaktorenanalyse mit AP3, das Design und die Umsetzung der Oberfläche für die Überwachungsfunktionen mit AP6, die Integration der umgesetzten visuellen Designs mit AP4 und anschließend die Evaluation mit den Expert*innen der Infektionsprävention/Hygiene.

Die unterschiedlichen IT-Infrastrukturen und rechtlichen Rahmenbedingungen an den verschiedenen Projektpartnerstandorten führen zu einem erheblichen Mehraufwand in der Umsetzung von entwickelten Lösungen in die Praxis. Geschieht die Integration in die verschiedenen Systeme erst spät oder am Ende des Entwicklungsprozesses, könnte der entscheidende Schritt der Umsetzung der Theorie in die Praxis sehr aufwendig werden.

*AP8 *(Ausrollen der entwickelten Demonstrator-Apps) verfolgt deshalb im Rahmen von RISK PRINCIPE einen konsequent agilen Ansatz zum Ausrollen der in den anderen Arbeitspaketen (AP3/AP4/AP7 und AP5/AP6/AP7) entwickelten Applikationen zur Infektions-Surveillance und Risikovorhersage. Durch eine frühe Einbindung aller beteiligten Parteien, unter anderem medizinisches Personal, IT-Administration, IT-Sicherheit, Datenschutz und Ethik und durch zeitnahe Integration in den Entwicklungsprozess können Schwierigkeiten frühzeitig erkannt und adressiert werden.

Dazu wird eine Entwicklungsumgebung geschaffen, welche die Vielfalt der an den verschiedenen Standorten anzutreffenden Rahmenbedingungen aufgreift und die prinzipielle Lauffähigkeit und Einsetzbarkeit der entwickelten Lösungen sicherstellt. In der späten Phase der Entwicklung müssen so nur noch Detailänderungen umgesetzt werden. Das Ziel dieses Arbeitspaketes ist, den Nutzen von den im Rahmen dieses Projektes entwickelten Apps zu maximieren. Die Umsetzung in die Praxis ist hierfür das wesentliche letzte Glied in der Kette der Entwicklungsarbeiten.

*AP9* (patient*innenzentrierte Reaktion und Intervention) umfasst die Entwicklung umfassender Datenmodelle, die Reaktionen und Interventionen der Infektionsprävention/Hygiene sowie Infektiologie auf ein erhöhtes individuelles HOB-Risiko und auf erhöhte HOB-Häufung abbilden. Ein gesteigertes individuelles bzw. patient*innengruppenspezifisches HOB-Risiko und ein Anstieg der Häufigkeit von HOB stellen dabei das zentrale Auslöserereignis dar, welches zum Beispiel aus den zu entwickelnden Applikationen der AP4 und AP6 von RISK PRINCIPE, aber auch aus existierenden Routinedaten entnommen werden kann.

Die geplanten Datenmodelle werden dazu in der Lage sein, die zielgerichteten Interventionen und Reaktionen in einer hoch standardisierten Weise umfassend und einfach durch die Anwender*innen zu erfassen. Dabei werden die Modelle die Möglichkeit bieten, bereits bestehende Infektionsschutzmaßnahmen auf Ebene von Patient*innen und Krankenhausstationen ebenso wie die konkret durchgeführte Intervention/Reaktion abzubilden.

Durch evidenzbasierte, vorstrukturierte und annotierte Interventionsmaßnahmen und -bündel, welche in den Datenmodellen hinterlegt sind, kann zudem das Fachpersonal lokal und zielgerichtet in der praktischen Arbeit unterstützt werden. Das Befüllen der Datenmodelle erfolgt durch benutzerfreundliche Datenerfassungsformulare, welche sich in die Visualisierungs- und Applikationslandschaft des Gesamtprojektes einfügen werden.

Die Entwicklung der Datenmodelle mit den zugehörigen Datenerfassungsformularen wird so konzipiert, dass die Modelle in die MeDICs der teilnehmenden universitären Klinikstandorte integriert werden können. Damit wird die technische Grundlage geschaffen, um standortübergreifende Analysen der entstehenden Datensätze durchführen zu können. Zusammenfassend zielt AP9 darauf ab, den Umgang mit einem gesteigerten individuellen HOB-Risiko und einer gesteigerten HOB-Häufigkeit im Sinne eines *Best-Practice*-Modells zu stärken.

## Netzwerkstruktur, Kooperation, Kollaboration, Patient*innenpartizipation, Nachhaltigkeit

In RISK PRINCIPE arbeitet ein multidisziplinäres Team von Spezialist*innen aus allen MII-Konsortien aus den Bereichen Medizininformatik, klinische Epidemiologie, Modellierung (sowohl aus der Statistik als auch aus der Informatik), Visual Engineering, Infektionsprävention/Hygiene, Infektiologie und Mikrobiologie sowie Surveillance zusammen. Wo sinnvoll, werden auch Patient*innen- und Verbraucher*innenperspektiven einbezogen.

Das Projekt wird durch die Kompetenzen von 5 weiteren Partnern unterstützt, u. a. 2 BG-Kliniken als assoziierte Partner, einem Klinikverbund mit 9 Akutkliniken und 2 Ambulanzen. Viele der RISK-PRINCIPE-Partner engagieren sich auch in leitender Position in verschiedenen Projekten des Netzwerks Universitätsmedizin (NUM), z. B. CODEX+, CollPan, GenSurV, MolTraX, NAP-KON, PREPARED, und ermöglichen somit koordinierte Ansätze, wissenschaftlichen Transfer und Zusammenarbeit.

Innerhalb der Infrastruktur entwickelte Konzepte werden grundsätzlich nach den Grundsätzen von *Open Source* oder *Open Access* offen verfügbar gemacht. Daher werden beide Demonstrator-Apps als *Open-Source*-Projekte (*GitHub*) zur Verbreitung und Nachnutzung durch interessierte Forscher*innen entwickelt und damit das bereits mit der SmICS-Anwendung (*Smart Infection Control System* [22]) erfolgreich verfolgte Modell fortgeführt.

Die besondere Wiederverwendbarkeit der generierten und gesammelten Daten für zukünftige Forschung wird durch die umfassende Umsetzung der FAIR-Prinzipien unter Einhaltung der EU Datenschutz-Grundverordnung gewährleistet, wie in der MII entwickelt. Soweit mit dem Datenschutz vereinbar, werden daher alle erhobenen Daten in etablierten öffentlichen Datenbanken zur Verfügung gestellt.

Durch die Kooperation mit europaweiten Surveillance-Initiativen und -projekten wird auf eine internationale Vernetzung geachtet. Da das RKI das *WHO AMR Surveillance and Quality Assessment Collaborating Center Network* koordiniert, geht die Reichweite der Entwicklung sogar über Europa hinaus.

## Fazit

RISK PRINCIPE ist ein komplexes, multidisziplinäres Projekt, das auf die Prävention und Risikoprädiktion nosokomialer Infektionen am Beispiel der HOB abzielt, um prototypisch innovative Lösungsansätze für die gegenwärtigen Herausforderungen im Gesundheitswesen zu erarbeiten.

Die verschiedenen AP konzentrieren sich auf Datenintegration, Risikoanalyse, digitale Tools und Surveillance-Methoden, um ein umfassendes System zur Identifizierung, Vorhersage und Reduktion nosokomialer Infektionen zu schaffen. Durch das RISK-PRINCIPE-Forschungsvorhaben könnten Patient*innen und Patient*innengruppen von spezifisch maßgeschneiderten Maßnahmen zur Prävention von Infektionen profitieren, was gleichzeitig zu einem effizienteren Einsatz von Ressourcen im Gesundheitswesen führen, die Patient*innensicherheit erhöhen und Kosten für den Krankenhausaufenthalt durch den gezielten spezifischen Einsatz preisintensiver Präventionsmaßnahmen (z. B. Chlorhexidin-Pflaster) und Vermeidung von Infektionen senken könnte.
